# Incidence and risk factors of monozygotic twinning following ART: analysis of 154 671 live births resulting from single embryo transfer

**DOI:** 10.1093/humrep/deaf121

**Published:** 2025-06-30

**Authors:** Repon C Paul, Christos A Venetis, Oisin Fitzgerald, Georgina M Chambers

**Affiliations:** National Perinatal Epidemiology and Statistics Unit (NPESU), Centre for Big Data Research in Health (CBDRH), University of New South Wales, Sydney, Australia; National Perinatal Epidemiology and Statistics Unit (NPESU), Centre for Big Data Research in Health (CBDRH), University of New South Wales, Sydney, Australia; Unit for Human Reproduction, 1st Department of Obstetrics and Gynaecology, School of Medicine, Faculty of Health Sciences, Aristotle University of Thessaloniki, Thessaloniki, Greece; National Perinatal Epidemiology and Statistics Unit (NPESU), Centre for Big Data Research in Health (CBDRH), University of New South Wales, Sydney, Australia; National Perinatal Epidemiology and Statistics Unit (NPESU), Centre for Big Data Research in Health (CBDRH), University of New South Wales, Sydney, Australia

**Keywords:** monozygotic twins, ART, single embryo transfer, blastocyst transfer, zygotic splitting

## Abstract

**STUDY QUESTION:**

What is the incidence of and risk factors for monozygotic twinning (MZT) following single embryo transfer (SET) in ART cycles in Australia and New Zealand?

**SUMMARY ANSWER:**

MZT occurred in 1.5% of live births following SET, with blastocyst transfer and fresh embryo transfer identified as key risk factors, while vitrified-thaw transfers were associated with a lower MZT risk.

**WHAT IS KNOWN ALREADY:**

ART has been associated with a higher incidence of MZT compared to natural conception. Previous studies have suggested younger maternal age, blastocyst culture, fresh embryo transfer, and certain ART techniques, such as assisted hatching and preimplantation genetic testing may elevate MZT risk. However, findings have been inconsistent, and with many prior studies underpowered and few reflecting contemporary ART practices.

**STUDY DESIGN, SIZE, DURATION:**

This retrospective cohort study analyzed data from 590 441 SET cycles conducted between 2009 and 2021 in Australia and New Zealand. The analysis included 154 671 live births following autologous SET cycles recorded in the Australian and New Zealand Assisted Reproductive Technology Database (ANZARD).

**PARTICIPANTS/MATERIALS, SETTING, METHODS:**

The study focused on autologous fresh and thawed SET cycles. MZT incidence was estimated by applying Weinberg’s differential rule, which assumes a 1:1 ratio of sex-concordant and sex-discordant dizygotic twins in the population of twins born following SET cycles. A multivariable logistic regression model with generalized estimating equations was used to identify risk factors for MZT, adjusting for potential misclassification of zygosity due to the absence of DNA confirmation.

**MAIN RESULTS AND THE ROLE OF CHANCE:**

The MZT rate was 1.5% among live births following SET. Blastocyst transfer was associated with a nearly 2-fold increase in MZT risk compared to cleavage-stage transfer (adjusted odds ratio [aOR] = 1.99, 95% CI: 1.71–2.31), and vitrified-thaw transfers had a lower MZT risk than fresh transfers (aOR = 0.87, 95% CI: 0.79–0.95). Sensitivity analyses supported these findings, with consistent MZT risk patterns across subgroups by maternal age, fertilization technique, and embryo transfer type (fresh/frozen).

**LIMITATIONS, REASONS FOR CAUTION:**

Zygosity estimation was based on Weinberg’s differential rule rather than DNA testing, which could lead to some misclassification. Additionally, the study lacked data on embryo quality, a variable with potential influence on MZT risk, and was limited to a retrospective design, potentially introducing treatment and information biases.

**WIDER IMPLICATIONS OF THE FINDINGS:**

This large-scale study identifies blastocyst transfer and fresh embryo transfer as significant MZT risk factor in ART, with potential implications for patient counseling and obstetric care. Future research should further investigate the mechanisms underlying these associations.

**STUDY FUNDING/COMPETING INTEREST(S):**

Funding was received from the Ferring Pharmaceuticals Pty Ltd as part of the Ferring FSANZ Leaders in Fertility Research and Education grant (to R.C.P.). The sponsors had no role in the design and conduct of the study; data collection, management, analysis, and interpretation; manuscript preparation, review, or approval; or the decision to submit for publication. FSANZ contracts National Perinatal Epidemiology and Statistics Unit (NPESU) of the University of New South Wales (UNSW) to prepare annual reports and benchmarking reports from the ANZARD: one of those datasets is used in this study. R.C.P. is a Research Fellow of the NPESU, UNSW; C.A.V. is affiliated with the NPESU, UNSW; G.M.C. is an employee of the UNSW and is the Director of the NPESU, UNSW. C.A.V., based at Aristotle University of Thessaloniki (Greece), is a member of the Executive Board of the Hellenic Society of Fertility and Sterility and serves as Senior Deputy of the Steering Committee for the SIG Reproductive Endocrinology of ESHRE. C.A.V. also reports lecture and advisory roles from Merck Ltd, Merck Sharpe & Dohme, Ferring, Organon, Gedeon-Richter, IBSA, Vianex, and Sonapharm; travel support from Merck Ltd, Merck Sharpe & Dohme, Ferring, Organon, Gedeon-Richter, and Vianex; and holds stock or stock options in Virtus Health Ltd, all outside the submitted work. O.F. reports funding from Ferring Pharmaceuticals Pty Ltd, unrelated to this study.

**TRIAL REGISTRATION NUMBER:**

N/A.

## Introduction

Multiple gestation pregnancies represent a significant complication of ART (Murray and Norman, 2014). While elective single embryo transfer (SET) is a key strategy to mitigate multiple gestation pregnancies in ART (Dessolle *et al.*, 2010), the risk of multiple births remains due to occurrences of early embryo splitting, leading to monozygotic twins (MZTs) (Vega *et al.*, 2018). Twins carry significantly increased risks of adverse perinatal outcomes, including preterm birth and higher perinatal and neonatal mortality, in comparison to singleton pregnancies (Glinianaia *et al.*, 2011; Obiechina *et al.*, 2011; Vasak *et al.*, 2017; Bellizzi *et al.*, 2018; Lin *et al.*, 2024). These risks are compounded in MZT cases by complications such as twin-to-twin transfusion syndrome, growth discordance, and congenital anomalies arising from shared placental structures (Benirschke, 1995).

Several studies, including a recent meta-analysis, have reported a higher incidence of MZT following ART, with rates between 0.97% and 2.35%, compared to the natural conception rate of 0.4% (Derom *et al.*, 1987; Tandberg *et al.*, 2007; Vitthala *et al.*, 2009; Busnelli *et al.*, 2019). Blastocyst culture, fresh embryo transfer, ICSI, preimplantation genetic testing (PGT), assisted hatching, and maternal age have been identified as possible risk factors for MZT after ART (Vitthala *et al.*, 2009; Mateizel *et al.*, 2016; Kamath *et al.*, 2020; Dallagiovanna *et al.*, 2021; Chen *et al.*, 2023). Notably, two recent meta-analysis highlighted blastocyst transfer as a significant contributor of MZT, each reporting pooled odds ratios above 2.0 (Hviid *et al.*, 2018; Busnelli *et al.*, 2019). However, the challenge in discerning associations arises from inconsistent findings, and many prior studies lacking the necessary power to effectively identify associations (Vitthala *et al.*, 2009; Mateizel *et al.*, 2016; Hviid *et al.*, 2018; Busnelli *et al.*, 2019). Moreover, older studies may not reflect current laboratory technologies and practices, potentially affecting the relevance of their findings on MZT incidence and risk factors in contemporary ART settings. Accurate estimation of MZT incidence and identification of relevant risk factors are essential for guiding patient counseling and clinical practice, allowing for avoidance of specific techniques that may elevate MZT risk, where possible, and increasing obstetric monitoring involving these procedures.

Australia and New Zealand collectively perform over 110 000 ART cycles each year representing one of the highest ART utilization rates globally, largely due to supportive public funding and no treatment limits based on female age or number of cycles offered (Harris *et al.*, 2016). Australia and New Zealand have also consistently achieved SET transfer rates above 90% since 2018, reaching 94.2% in 2022 (Newman *et al.*, 2024). The region maintains a comprehensive ART registry, the Australian and New Zealand Assisted Reproductive Technology Database (ANZARD) that records detailed patient and treatment information on every ART cycle undertaken in Australia and New Zealand (Chambers *et al.*, 2017; Paul *et al.*, 2020; Newman *et al.*, 2024). This unique regional context provides an ideal setting to evaluate a wide range of patient and ART treatment factors that may influence the risk of MZTs. The aim of this study was to investigate the incidence and the risk factors for MZTs following SET in the context of ART treatment.

## Materials and methods

### Data source and study population

Thirteen years of ART treatment cycles (2009–2021) involving autologous (patient’s own oocytes) fresh and thawed cycles were extracted from ANZARD. Records of all autologous thawed cycles were linked to the corresponding episodes of ovarian stimulation for each woman, allowing the identification of patient and treatment characteristics of women at the time of ovarian stimulation. The scope of this study was limited to the examination of clinical pregnancies and live births resulting from autologous SET cycles. Cycles involving multiple embryo transfers and those utilizing donor oocytes or sperm were excluded. The study protocol was approved by the Human Research Ethics Advisory Panel, University of New South Wales (Protocol No. HC220086).

### Definitions of outcomes

A clinical pregnancy was defined as one that met at least one of the following criteria: ongoing at 20 weeks, ultrasound evidence of an intrauterine sac (with or without a fetal heartbeat), identification of chorionic villi in the products of conception after spontaneous, medical or surgical evacuation of the uterus, or confirmation of an ectopic pregnancy via laparoscopy or ultrasound (Newman *et al.*, 2024). A live birth was defined as the delivery of a liveborn infant of at least 20 weeks gestation or a minimum birth weight of 400 grams. In this study, any sex-discordant twins following SET were assumed to result from dizygotic twinning (DZT), likely due to concurrent natural conception, as MZTs cannot be sex-discordant.

In the absence of zygosity confirmation through DNA testing, Weinberg’s differential rule was applied to estimate the incidence of MZT births, a widely used approach in ART literature. According to this rule, the number of sex-concordant DZTs is assumed to equal the number of sex-discordant DZTs, provided that the population sex ratio is balanced (male/female sex ratio = 1) (Pflüger, 1901). In accordance with Weinberg’s rule, the number of MZT births following SET was calculated by subtracting the count of sex-discordant twin births from the count of sex-concordant twin births observed in our study (Fellman and Eriksson, 2006). Sex-concordant triplets and higher-order births were included in the count of sex-concordant twins for the purpose of this analysis.

### Statistical analysis

We conducted a comparison of the patient and treatment characteristics between singleton births and sex-concordant twin births resulting from SETs. Categorical characteristics were compared using chi-squared tests, while continuous characteristics were compared using the Wilcoxon rank-sum test.

#### Multivariable analyses to identify risk factors for MZT

We employed a multivariable logistic regression model with a generalized estimating equation to identify risk factors for MZT among live births resulting from SET cycles, accounting for within-subject correlation due to repeated cycles from the same individual. Since not all sex-concordant twin births in our study are MZTs and some may be sex-concordant DZTs, we utilized an adjustment method outlined in the STRATOS guidance document on measurement error and misclassification of variables in observational studies (Shaw *et al.*, 2020) to address this misclassification. This was necessary because zygosity confirmation through DNA testing was not available. Using likelihood-based adjustments, this adjustment corrects for misclassification errors directly within the model by incorporating the known sensitivity (true MZT positive) and specificity (true MZT negative) of the outcome measure.

Covariates included in the model were selected based on previously reported risk factors of MZT in the literature and the available data in ANZARD. The model included the following covariates: the age of women at the time of oocyte retrieval, previous pregnancy, cause of infertility, number of oocytes retrieved, fertilization techniques (IVF/ICSI), stage of embryo transfer (cleavage/blastocyst), type of embryo transfer (fresh/frozen), PGT, assisted hatching, and treatment year.

#### Subgroup analyses

Given previous research indicating an increased risk of MZTs after blastocyst embryo transfer, we conducted several subgroup analyses to examine whether the association between blastocyst transfer and MZTs varies across different factors: women’s age at oocyte retrieval, type of embryo transfer (fresh/frozen), fertilization technique, treatment year, PGT, and assisted hatching. For each of the sub-groups, we calculated the adjusted odds ratio (aOR) of MZTs following SET in women who underwent blastocyst stage embryo transfer compared to those who underwent cleavage stage transfer. Each subgroup analysis was conducted separately using a multi-variable logistic regression model with generalized estimating equations. All the covariates used in the full model were also included in each subgroup analysis for comprehensive assessment and comparison. For example, to explore whether the association between blastocyst transfer and MZTs varies by ‘women age at oocyte retrieval’, we conducted four separate models for age groups <30, 30–34, 35–39, and ≥40 years, each adjusting for factors included in the main model, including previous pregnancy, cause of infertility, number of oocytes retrieved, fertilization technique (IVF/ICSI), type of embryo transfer (fresh/frozen), PGT, assisted hatching, and treatment year.

#### Sensitivity analysis

In ART treatment, surgical sperm retrieval is employed when sperm are absent upon ejaculation or when the male partner is unable to ejaculate. Given this, it is most likely that sex-concordant twin births born to couples where the male patient requires surgical sperm retrieval are indeed MZTs, as natural conception is less likely when sperm must be collected surgically. Hence, we performed a sensitivity analysis by restricting the analysis to live births where sperm retrieval was performed surgically. This allowed us to examine whether risk factors for sex-concordant twin births differ when all sex-concordant twin births are likely to be MZTs.

### Ethics approval

This study was approved by the University of New South Wales Human Research Ethics Advisory Panel (reference, HC220086). It used non-identifiable secondary data, with patient consent originally obtained for its use in research.

## Results

**Figure 1.**
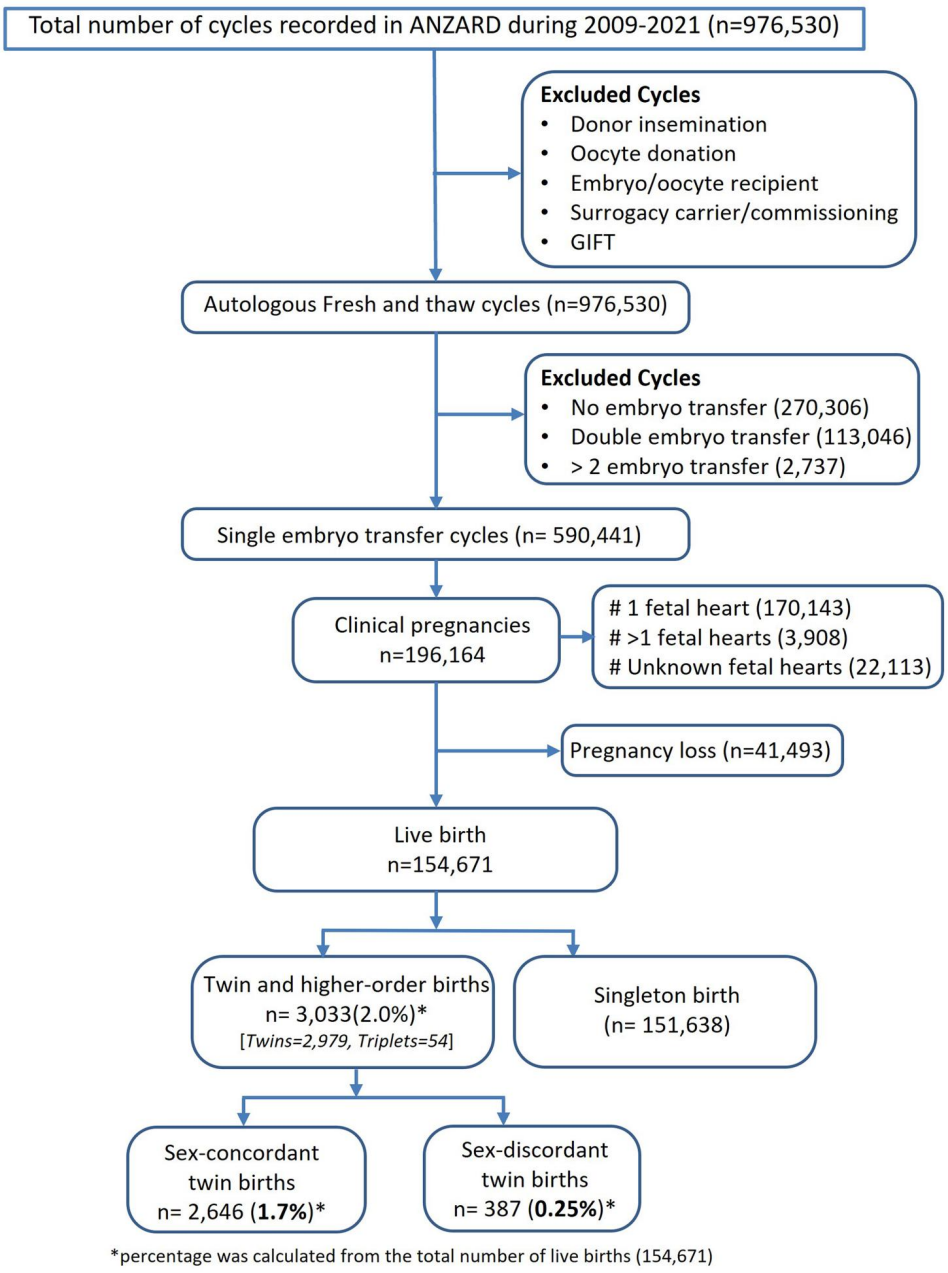
Flow chart of estimating the rate of multifetal pregnancies and monozygotic twin births after single embryo transfer in Australia and New Zealand, 2009–2021.

Between 2009 and 2021, a total of 590 441 SETs were performed in Australia and New Zealand, resulting in 196 164 clinical pregnancies (Fig. 1). Among the clinical pregnancies, the number of fetuses was unknown for 22 113 cases. Of the clinical pregnancies where the number of fetuses is known, 2.2% (3908/174 051) were multifetal pregnancies.

From the clinical pregnancies, 154 671 resulted in a live birth, and of which 2.0% (3033/154 671) were twin births (Fig. 1). Among the twins born, 51.7% were male. The sex-concordant twin birth rate was 1.7% (2646/154 671) and the sex-discordant rate was 0.3% (387/154 671). When applying Weinberg’s differential rule, the rate of MZT births was determined to be 1.5% (2259/154 671) and the rate of DZT births was 0.5% (774/154 671). The rate of MZT following SET varied over time ranging from 1.9% in 2009 (among 8377 live births) and 1.2% in 2018 (among 13 571 live births); in 2021, the rate stood at 1.5% (among 17 902 live births), representing the latest year analyzed in this study.

Among twin births, 12.8% (387/3033) were sex-discordant, corresponding to an estimated total DZT proportion of 25.5% (774/3033) based on Weinberg’s rule. Applying this estimate to all multifetal pregnancies following SET suggests an overall MZT pregnancy rate of ∼1.7% (2911/174 051).

Table 1 presents the patient and treatment characteristics of singletons and sex-concordant twin births from SET. The median maternal age at oocyte retrieval was 33 years in both groups, with a small but significant difference in age distribution (*P* = 0.032), notably with a higher proportion of women aged 30–34 years among twin births (44.1%) compared to singletons (41.3%). Previous pregnancy status was similar between groups (*P* = 0.383). Infertility causes, such as male-only or female factors such as endometriosis and tubal disease, were also comparable, with no significant differences observed. The median number of oocytes retrieved was 11 in both groups (*P* = 0.955). Fertilization techniques and PGT rates showed no significant differences (*P* = 0.067 and *P* = 0.529, respectively). However, blastocyst-stage embryo transfers were more common among twins (90.7%) than singletons (85.8%) (*P* < 0.001), and fresh embryo transfers were more frequent in twin births (47.5%) compared to singletons (45.5%, *P* = 0.025).

**Table 1.** Patient and treatment characteristics of singleton births and sex-concordant twin births after single embryo transfer in Australia and New Zealand, 2009–2021.

Characteristics	Singleton births (N = 151 638)		Sex-concordant twin births (N = 2646)		*P*-value*
	n	%	n	%	
Female age at the time of oocyte retrieval (years); median (interquartile range, IQR)	33 (30–36)		33 (30–36)		**0.032**
<30	27 713	18.3	478	18.1	
30–34	62 702	41.3	1166	44.1	
35–39	51 025	33.6	843	31.9	
40–44	10 056	6.6	157	5.9	
>44	111	0.1	1	0.0	
Missing	31	0.0	1	0.0	
Previous pregnancy					0.383
No	107 304	70.8	1893	71.5	
Yes	44 334	29.2	753	28.5	
Cause of infertility					
Male-only	28 479	18.8	495	18.7	0.924
Female factor					
Tubal disease only	7793	5.1	120	4.5	0.163
Endometriosis only	11 078	7.3	188	7.1	0.694
Both tubal and endometriosis	1754	1.2	34	1.3	0.541
Other female factor only	27 971	18.4	494	18.7	0.769
Both male–female factors	16 964	11.2	295	11.1	0.951
Unexplained	35 531	23.4	658	24.9	0.084
Not stated	22 068	14.6	362	13.7	0.207
Number of oocytes retrieved, median (IQR)	11 (7–16)		11 (7–16)		0.955
1–4	13 119	8.7	207	7.8	
5–9	45 035	29.7	795	30.0	
10–14	43 197	28.5	776	29.3	
15–19	25 221	16.6	431	16.3	
20 or more	20 614	13.6	349	13.2	
Missing	4452	2.9	88	3.3	
Fertilization technique					0.067
IVF	56 332	37.1	1022	38.6	
ICSI	90 275	59.5	1520	57.4	
Missing	5031	3.3	104	3.9	
Stage of embryo transfer					**<0.001**
Cleavage	21 529	14.2	247	9.3	
Blastocyst	130 109	85.8	2399	90.7	
Type of embryo transfer					**0.025**
Fresh	68 967	45.5	1256	47.5	
Thaw—slow frozen	12 172	8.0	229	8.7	
Thaw—vitrified	70 499	46.5	1161	43.9	
Preimplantation genetic testing (PGT)					0.529
No	141 728	93.5	2465	93.2	
Yes	9910	6.5	181	6.8	
Assisted hatching without PGT					0.655
No	142 442	93.9	2480	93.7	
Yes	9196	6.1	166	6.3	

* Categorical variables were compared using chi-squared tests, while continuous variables were compared using the Wilcoxon rank-sum test.

*P*-values in bold indicate statistical significance at *P* < 0.05.

### Risk factors for MZT

The multivariable analysis, adjusted for misclassification errors, identified blastocyst and fresh embryo transfers, as well as the year of treatment, as independent risk factors for MZT births among the birth events (Table 2). The adjusted odds of MZTs were approximately twice as high in instances involving blastocyst embryo transfer compared to those involving cleavage stage embryo transfer (aOR = 1.99, 95% CI: 1.71–2.31). The likelihood of MZT births decreased by 13% for vitrified thaw transfers compared to cycles involving fresh transfers (aOR = 0.87; 95% CI: 0.79–0.95). Slow-frozen thaw transfers exhibited similar odds of MZT births as fresh transfers. Furthermore, the risk of MZT births exhibited a significant decline over time. Specifically, in the years 2019–2021 compared to the reference period of 2009–2011, the adjusted odds decreased by 22% (aOR = 0.78; 95% CI: 0.70–0.88). Other factors, including female age at oocyte retrieval, previous pregnancy, infertility causes, number of oocytes retrieved, fertilization technique (IVF vs ICSI), PGT, and assisted hatching, did not significantly impact MZT birth risk after SET.

**Table 2.** Risk factors of ART monozygotic twin births after single embryo transfers in Australia and New Zealand, 2009–2021.

Characteristics	Unadjusted odds ratio	95% CI		*P*-value	Adjusted^¥^ odds ratio	95% CI		*P*-value
Female age at the time of oocyte retrieval (years)								
<30	Ref				Ref			
30–34	1.08	0.97	1.20	0.167	1.08	0.97	1.21	0.164
35–39	0.96	0.86	1.07	0.462	0.95	0.84	1.06	0.358
≥40	0.90	0.75	1.08	0.260	0.90	0.75	1.09	0.271
Previous pregnancy								
No	Ref				Ref			
Yes	0.97	0.89	1.05	0.439	0.98	0.89	1.07	0.604
Cause of infertility*								
Male-only	1.00	0.90	1.10	0.926	1.00	0.86	1.15	0.978
Female factor								
Tubal disease only	0.88	0.73	1.05	0.161	0.84	0.67	1.04	0.111
Endometriosis only	0.97	0.84	1.13	0.698	0.96	0.80	1.15	0.666
Both tubal and endometriosis	1.11	0.79	1.57	0.540	0.99	0.68	1.44	0.946
Other female factor only	1.01	0.92	1.12	0.777	1.01	0.88	1.17	0.877
Both male–female factors	1.00	0.88	1.13	0.955	0.97	0.83	1.14	0.734
Unexplained	1.08	0.99	1.18	0.087	1.04	0.91	1.20	0.515
Number of oocytes retrieved								
1–4	Ref				Ref			
5–9	1.12	0.96	1.31	0.153	0.99	0.84	1.15	0.870
10–14	1.14	0.98	1.33	0.100	0.98	0.83	1.15	0.768
15–19	1.08	0.92	1.28	0.343	0.92	0.78	1.10	0.375
20 or more	1.07	0.90	1.28	0.420	0.93	0.78	1.12	0.476
Fertilization technique								
IVF	Ref				Ref			
ICSI	0.93	0.86	1.01	0.071	0.93	0.85	1.02	0.109
Stage of embryo transfer								
Cleavage	Ref				Ref			
Blastocyst	1.61	1.41	1.83	**<0.001**	1.99	1.71	2.31	**<0.001**
Type of embryo transfer								
Fresh	Ref				Ref			
Thaw—slow frozen	1.04	0.90	1.19	0.634	1.05	0.89	1.25	0.532
Thaw—vitrified	0.91	0.84	0.98	**0.017**	0.87	0.79	0.95	**0.002**
Preimplantation genetic testing (PGT)								
No	Ref				Ref			
Yes	1.05	0.90	1.22	0.528	1.06	0.89	1.26	0.528
Assisted hatching without PGT								
No	Ref				Ref			
Yes	1.09	0.96	1.24	0.164	1.12	0.97	1.29	0.122
Treatment year								
2009–2012	Ref				Ref			
2013–2015	0.92	0.83	1.03	0.160	0.90	0.80	1.02	0.096
2016–2018	0.81	0.73	0.91	**<0.001**	0.77	0.68	0.87	**<0.001**
2019–2021	0.84	0.76	0.94	**0.001**	0.78	0.70	0.88	**<0.001**

* The reference category for cause of infertility was ‘not present’, e.g. for male-only infertility, the reference category equated to ‘male infertility not present’.

¥ Adjusted for age of women at the time of oocyte retrieval, previous pregnancy, cause of infertility, number of oocytes retrieved, fertilization techniques (IVF/ICSI), stage of embryo transfer (cleavage/blastocyst), type of embryo transfer (fresh/frozen), PGT, assisted hatching, and treatment year.

*P*-values in bold indicate statistical significance at *P* < 0.05.

### Subgroup analyses

Table 3 presents a subgroup analysis of aORs for MZT births following SET, showing consistently higher MZT birth rates with blastocyst-stage compared to cleavage-stage transfers across most subgroups. In the subgroup of female age at oocyte retrieval, blastocyst transfers increased the odds of MZT births significantly across all ages, with a 50% increase for women under 30 (aOR = 1.5, *P* = 0.013) and even higher odds for ages 30–34, 35–39, and ≥40 years (aORs of 2.3, 1.7, and 2.4, respectively). For embryo transfer type, fresh blastocyst transfers doubled MZT odds (aOR = 2.2, *P* < 0.001), while slow-frozen thaw transfers also showed increased risk (aOR = 1.5, *P* = 0.040); vitrified-thaw transfers showed no significant difference. Both IVF (aOR = 2.1) and ICSI (aOR = 1.9) with blastocyst transfers were associated with elevated MZT odds. MZT incidence remained consistently higher in blastocyst transfers compared to cleavage-stage transfers throughout the treatment period. However, in cycles using PGT or assisted hatching, no significant differences were observed between blastocyst and cleavage-stage transfers.

**Table 3.** Adjusted odds ratio for monozygotic twin births following single embryo transfers with cleavage and blastocyst-stage embryo transfer.

Groups	Cleavage stage ET*	Blastocyst stage ET	Adjusted odds ratio^¥^	*P*-value
Female age at the time of oocyte retrieval (years)				
<30	56/4193 = 1.3%	422/23 998 = 1.8%	1.5 (1.1–2.1)	0.013
30–34	92/8781 = 1%	1074/55 087 = 1%	2.3 (1.8–3.0)	<0.001
35–39	85/7314 = 1.2%	758/44 555 = 1.7%	1.7 (1.4–2.2)	<0.001
≥40	13/1482 = 0.9%	145/8842 = 1.6%	2.4 (1.3–4.4)	0.005
Type of embryo transfer				
Fresh transfer cycles	145/15 440 = 0.9%	1111/54 783 = 2%	2.2 (1.8–2.6)	<0.001
Thaw—slow frozen	82/4906 = 1.7%	147/7495 = 2%	1.5 (1.0–2.1)	0.040
Thaw—vitrified	20/1430 = 1.4%	1141/70 230 = 1.6%	1.2 (0.8–1.9)	0.773
Fertilization technique				
IVF	76/7410 = 1%	946/49 943 = 1.9%	2.1 (1.6–2.6)	<0.001
ICSI	137/12 831 = 1.1%	1383/78 963 = 1.8%	1.9 (1.6–2.3)	<0.001
Preimplantation genetic testing (PGT)				
No	245/21 678 = 1.1%	2220/122 515 = 1.8%	1.9 (1.7–2.3)	<0.001
Yes	2/98 = 2%	179/9993 = 1.8%	1.7 (0.4–7.2)	0.482
Assisted hatching without PGT				
No	241/21 519 = 1.1%	2134/118 166 = 1.8%	2.0 (1.7–2.3)	<0.001
Yes	6/257 = 2.3%	265/14 342 = 1.8%	1.0 (0.4–2.3)	0.994
Treatment year				
2009–2012	141/9876 = 1.4%	516/24 125 = 2.1%	1.6 (1.3–2.0)	<0.001
2013–2015	61/5871 = 1%	530/27 282 = 1.9%	1.9 (1.5–2.6)	<0.001
2016–2018	23/3470 = 0.7%	587/35 339 = 1.7%	2.8 (1.8–4.3)	<0.001
2019–2021	22/2559 = 0.9%	766/45 762 = 1.7%	2.1 (1.4–3.3)	0.001

* Reference category.

¥ Models were adjusted for female age at the time of oocyte retrieval, previous pregnancy, cause of infertility, number of oocytes retrieved, fertilization technique, stage of embryo transfer, type of embryo transfer, PGT, assisted hatching without PGT, and the year of treatment.

Separate models were run for each of the categories. For example, for fertilization technique, data were divided into two groups (IVF and ICSI), and the models were run separately to calculate the odds ratio for monozygotic twins for each category.

### Sensitivity analyses based on source of sperm

In our study, among the 7202 live births where sperm retrieval occurred through surgical procedures, there were 122 (1.7%) instances of sex-concordant twin births and only two (0.03%) instances of sex-discordant twin births (Supplementary Table S1). In the sensitivity analysis, we found no significant changes in the odds ratios of the risk factors associated MZT births when the analysis was restricted to cases involving surgical sperm retrieval. Specifically, we observed that the odds of MZTs were 1.9 times higher in blastocyst-stage embryo transfer compared to cleavage-stage embryo transfer (aOR = 1.9; 95% CI: 1.1–3.5). Additionally, the likelihood of MZTs was 0.6 times lower in vitrified thaw cycles compared to fresh embryo transfer cycles (aOR = 0.4; 95% CI: 0.3–0.7).

## Discussion

This study presents a comprehensive analysis of MZT incidence and risk factors following SET in a large ART cohort from Australia and New Zealand over a 13-year period. Despite the use of SET, twin births occurred in 2% of cases, with an estimated MZT incidence of 1.5%. Our findings highlight blastocyst-stage embryo transfer as a significant risk factor, almost doubling the likelihood of MZT compared to cleavage-stage transfer. However, the risk of MZT has decreased over time, potentially reflecting shifts in ART protocols that were not measured in our study. As the largest and most contemporary study of its kind to date, this analysis evaluated over 590 000 SET cycles, resulting in over 196 000 clinical pregnancies, 154 671 live births, and 3033 twin births.

Similar to earlier research, our study illustrates that even with SET, the potential for a multiple pregnancy persists, with a rate approximately four times higher than the MZT rate observed in natural conception (0.4%) (Bulmer, 1970; Derom *et al.*, 1987; Tandberg *et al.*, 2007; Vitthala *et al.*, 2009). A principal finding in our study is the strong association between blastocyst-stage embryo transfer and increased MZT risk, a factor that persists across various subgroups, including maternal age, fertilization technique, and embryo transfer type. This finding aligns with meta-analyses by Busnelli *et al.* (2019) and Hviid *et al.* (2018). Although the precise mechanism linking blastocyst transfer to MZT remains uncertain, extended culture conditions are posited to impact the zona pellucida, leading to splitting of the inner cell mass (Behr *et al.*, 2000; Vitthala *et al.*, 2009; Ikemoto *et al.*, 2018). The extended exposure to specific culture media may harden the zona, increasing MZT susceptibility. Furthermore, variations in calcium concentrations, glucose-induced apoptosis, and growth factor levels may destabilize intracellular bonds or influence cellular signaling, affecting the likelihood of twinning (Ménézo and Sakkas, 2002; Papanikolaou *et al.*, 2010; Sotiroska *et al.*, 2015; Ikemoto *et al.*, 2018). Additionally, blastocyst characteristics and sensitivity to lab conditions may play a role, with hypotheses suggesting increased twinning susceptibility due to mechanical manipulation or transient changes in temperature or pH during monitoring or embryo transfer (Luke *et al.*, 2014).

Our data also reveal a lower MZT risk in vitrified-thaw embryo transfers compared to fresh transfers. While several studies have similarly reported an elevated MZT risk with fresh embryo transfers compared to thawed transfers (Mateizel *et al.*, 2016; Vega *et al.*, 2018; Chen *et al.*, 2023), Ikemoto *et al.* (2018) reported thawed embryo transfer as a risk factor of MZT. These contrasting findings may be due to differences in freezing techniques, study populations, and the covariates considered in risk estimation models. However, our study is the first to specifically show that vitrified-thaw transfers—not slow-frozen transfers—have a reduced MZT risk relative to fresh transfers. The higher MZT rate in fresh transfers suggests that factors intrinsic to the fresh embryo transfer process, such as hormonal variations post-ovarian stimulation (Liu *et al.*, 2020; Khattak *et al.*, 2022), may contribute to increased twinning. Fresh embryo transfers occur shortly after ovarian stimulation, which can create a hyperstimulated endometrial environment (Zaat *et al.*, 2021). This hyperstimulation, characterized by elevated levels of estrogen and progesterone, may disrupt the delicate balance of endometrial receptivity or alter implantation dynamics (Ma *et al.*, 2003; Liang *et al.*, 2018), potentially increasing the likelihood of zygotic splitting. Mateizel *et al.* argued that these endometrial disparities during transfers following ovarian stimulation could explain the heightened MZT occurrence, warranting further investigation into this hypothesis (Mateizel *et al.*, 2016). Nevertheless, it is intriguing to note that vitrified freezing, the most recent and widely adopted technology for embryo preservation, also demonstrates a lower risk of MZT.

While blastocyst transfer emerged as a robust MZT risk factor, we found no significant independent associations with other variables previously suggested to influence MZT rates, such as maternal age, fertilization technique (IVF vs ICSI), assisted hatching, and PGT (Song *et al.*, 2017; Hviid *et al.*, 2018; Busnelli *et al.*, 2019; Kamath *et al.*, 2020; Chen *et al.*, 2023). These discrepancies may arise from different methodological designs in prior studies, including availability of covariates for statistical adjustments and smaller sample sizes that might have precluded in-depth analyses and detection of true associations, whereas the substantial dataset utilized in our study provided adequate power for detecting true associations. The incidence of MZTs declined over time, with significantly lower odds observed in recent years compared to 2009–2012, despite adjustments for known risk factors. While the exact reasons remain unclear, advances in culture media, improved lab conditions, and precise control of pH, oxygen levels, and temperature may have reduced zygotic splitting (Gardner and Kelley, 2017; Ng *et al.*, 2018; Brouillet *et al.*, 2021). These findings highlight the complex and multifactorial nature of MZT in ART, suggesting that further investigation into individual factors, recent advancements in ART technologies, and their interactions with embryo development stages is essential.

We acknowledge certain limitations in our study. First, zygosity could not be confirmed through DNA testing, and MZT incidence was instead estimated using Weinberg’s differential rule (Pflüger, 1901), a widely accepted statistical approximation for large population-based cohort studies (Osianlis *et al.*, 2014; Vega *et al.*, 2018). This approach has been validated in similar contexts and is unlikely to substantially affect our MZT incidence estimates (Fellman and Eriksson, 2006; James, 2007), given the near-balanced population sex ratio in Australia (51% male vs 49% female babies) (Australian Institute of Health and Welfare (AIHW), 2024), which aligns with Weinberg’s rule assumptions. However, the inability to specifically identify the sex-concordant DZT births (12.8% of twin births) due to the lack of zygosity testing may influence our risk factor analysis for MZT. To mitigate this limitation, we adjusted our logistic regression model for the sensitivity and specificity of MZT identification, thereby minimizing potential misclassification errors through the model itself. Furthermore, in our sensitivity analysis, where we restricted the analysis to live births resulting from couples where the male patient required surgical sperm retrieval procedures, the identified risk factors for MZT remained unchanged, reinforcing the robustness of our findings. This consistency suggests that the observed associations between MZT incidence and factors like blastocyst transfer stage and embryo transfer type are likely reliable, even in the context of potential misclassification. Second, the ANZARD registry does not collect gestational sac data, which limits the ability to distinguish true singleton pregnancies from those that may have initially involved early fetal loss (vanishing twin syndrome) (Gjerris *et al.*, 2012; Ikemoto *et al.*, 2018). Although we reported fetal numbers in clinical pregnancies and estimated the rate of monozygotic splitting accordingly, the absence of gestational sac information may have led to under-ascertainment of some monozygotic splitting events. However, as the primary aim of our study was to estimate the incidence and risk factors for liveborn MZT, rather than the biological incidence of zygotic splitting, this limitation is unlikely to materially affect the primary outcome. Third, we lacked information on embryo quality, which could influence MZT. However, the literature presents conflicting perspectives on the relationship between embryo quality and MZT incidence (Franasiak *et al.*, 2015; Otsuki *et al.*, 2016; Hviid *et al.*, 2018). Fourth, a significant number of clinical pregnancies had unknown fetal heart counts. This did not affect the calculation of MZT incidence (nor its risk factors), as MZTs are determined from live births which was the aim of the study. However, we recognize that missing fetal heart information would affect the reported percentage of multifetal pregnancies. The absence of fetal count information on ultrasound is likely due to incomplete reporting or follow-up, particularly in cases of early pregnancy loss or pregnancies without ultrasound confirmation at the time of the first scan. Finally, unmeasured patient-specific factors, such as uterine environment or lifestyle factors not available in our dataset, may contribute to unobserved heterogeneity in our results.

In conclusion, this study provides robust evidence that while SET is the key strategy to reduce multiple pregnancies in ART, MZT remains a notable risk, particularly with blastocyst and fresh embryo transfers, whereas vitrified-thaw transfers are associated with a lower risk of MZT. With the increasing adoption of extended embryo culture and blastocyst transfer in modern ART practice, it is essential to acknowledge the risk of MZT, ensuring that clinicians carefully evaluate embryo transfer strategies based on individual patient risk factors. Women with high obstetric risk, such as those with severe endometriosis or undergoing frozen embryo transfer with hormone replacement therapy, should receive personalized counseling, and SET should be the norm in all but exceptional cases to minimize the risks associated with multiple pregnancies. The comparatively lower MZT risk with vitrified-thaw transfer may be considered by clinicians and patients when making decisions on the embryo transfer strategy, particularly in cases where minimizing twin risk is a priority. As the field progresses, optimizing laboratory practices, refining culture media formulations, and exploring genetic factors associated with twinning may lead to more personalized ART approaches, reducing the likelihood of multiple pregnancies while maintaining ART efficacy (Swain, 2010; Mantikou *et al.*, 2013; Luke *et al.*, 2014).

## Supplementary Material

deaf121_Supplementary_Table_S1

## Data Availability

The data underlying this article cannot be shared publicly due to patient privacy concerns.
