# Supplementary material for: Incidence and risk factors of monozygotic twinning following ART: analysis of 154 671 live births resulting from single embryo transfer
Source: Hum Reprod. 2025 Jun 30;40(9):1744–52. doi: 10.1093/humrep/deaf121 (PMC12408896; doi:10.1093/humrep/deaf121)
Supplement: deaf121_Supplementary_Table_S1 [file deaf121_supplementary_table_s1.pdf]

Human Reproduction, 2025, 1–1
https://doi.org/10.1093/humrep/deaf121
Supplementary Data

Supplementary Table S1. Sensitivity analysis: risk factors of ART monozygotic twin births after single embryo transfers with surgically retrieved sperm in Australia and New Zealand, 2009–2021.

Characteristics	Adjusted[¥] odds ratio	95% CI		P-value
Female age at the time of oocyte retrieval (years)				
<30	Ref			
30–34	0.96	0.61	1.50	0.841
35–39	0.62	0.37	1.05	0.074
≥40	0.57	0.20	1.65	0.297
Previous pregnancy				
No				
Yes	1.00	0.66	1.51	0.994
Cause of infertility*				
Male-only	1.40	0.59	3.30	0.445
Female factor				
Tubal disease only	3.53	0.40	31.35	0.258
Endometriosis only	2.72	0.65	11.29	0.169
Both tubal and endometriosis	1.00			
Other female factor only	0.50	0.14	1.81	0.288
Both male–female factors	1.43	0.57	3.56	0.446
Unexplained	0.77	0.26	2.29	0.641
Not stated				
Number of oocytes retrieved, median (interquartile range)				
1–4	Ref			
5–9	0.67	0.32	1.40	0.288
10–14	0.99	0.48	2.01	0.968
15–19	0.59	0.26	1.34	0.208
20 or more	0.78	0.33	1.82	0.560
Stage of embryo transfer				
Cleavage	Ref			
Blastocyst	1.95	1.08	3.51	**0.026**
Type of embryo transfer				
Fresh	Ref			
Thaw—slow frozen	1.38	0.67	2.85	0.388
Thaw—vitrified	0.41	0.26	0.66	**<0.001**
Preimplantation genetic testing (PGT)				
No	Ref			
Yes	2.22	0.91	5.46	0.081
Assisted hatching without PGT				
No	Ref			
Yes	0.96	0.45	2.06	0.914
Treatment year				
2009–2012	Ref			
2013–2015	0.98	0.59	1.61	0.930
2016–2018	0.49	0.26	0.92	**0.026**
2019–2021	1.13	0.69	1.85	0.635

* The reference category for cause of infertility was 'not present', e.g. for male-only infertility, the reference category equated to 'male infertility not present'.
¥ Adjusted for age of women at the time of oocyte retrieval, previous pregnancy, cause of infertility, number of oocytes retrieved, stage of embryo transfer (cleavage/blastocyst), type of embryo transfer (fresh/frozen), PGT, assisted hatching, and treatment year.
P-values in bold indicate statistical significance at $P < 0.05$.

© The Author(s) 2025. Published by Oxford University Press on behalf of European Society of Human Reproduction and Embryology.
This is an Open Access article distributed under the terms of the Creative Commons Attribution License (https://creativecommons.org/licenses/by/4.0/), which permits unrestricted reuse, distribution, and reproduction in any medium, provided the original work is properly cited.
Human Reproduction, 2025, 00, 1–9
https://doi.org/10.1093/humrep/deaf121
Original Article
